# Supplementary material for: New Benzimidazothiazolone Derivatives as Tyrosinase Inhibitors with Potential Anti-Melanogenesis and Reactive Oxygen Species Scavenging Activities
Source: Antioxidants (Basel). 2021 Jul 5;10(7):1078. doi: 10.3390/antiox10071078 (PMC8301170; doi:10.3390/antiox10071078)
Supplement: Supplementary file 1 [file antioxidants-10-01078-s001.zip › antioxidants-1265677-supplementary.pdf]

Supporting Information

for

New benzimidazothiazolone derivatives as tyrosinase inhibitors with potential anti-melanogenesis and reactive oxygen species scavenging activities

Hee Jin Jung, Dong Chan Choi, Sang Gyun Noh, Heejeong Choi, Inkyu Choi, Il Young Ryu, Hae Young Chung* and Hyung Ryong Moon*

College of Pharmacy, Pusan National University, Busan 46241, Republic of Korea

**Correspondence: hyjung@pusan.ac.kr (Hae Young Chung),*

mhr108@pusan.ac.kr (Hyung Ryong Moon)

General experimental methods

All reagents were obtained commercially and used without further purification. Thin layer chromatography (TLC) and column chromatography were conducted using Merck precoated 60F245 plates and MP Silica 40-63, 60 Å, respectively. Mass data were obtained in ESI negative and positive modes on an Expression CMA spectrometer (Advion Ithaca, NY, USA). Nuclear magnetic resonance (NMR) spectra were recorded on a Varian Unity INOVA 400 spectrometer or a Varian Unity AS500 spectrometer (Agilent Technologies, Santa Clara, CA, USA) for ^{1}H NMR (400 and 500 MHz) and for ^{13}C NMR (100 MHz). DMSO-d_6 and $CDCl_3$ were used as an NMR solvent for NMR samples. The coupling constant (*J*) and chemical shift values were measured in hertz (Hz) and parts per million (ppm), respectively. The following abbreviations are used in the analysis of ^{1}H NMR data: s (singlet), brs (broad singlet), d (doublet), dd (doublet of doublets), t (triplet), q (quartet), and m (multiplet).

2-((1*H*-Benzo[*d*]imidazol-2-yl)thio)acetic acid (14)

To a stirred solution of 2-mercaptobenzimidazole (5.00 g, 33.29 mmol) in acetic acid (50 mL) were added bromoacetic acid (6.90 g, 49.66 mmol) and NaOAc (8.20 g, 99.96 mmol) and the reaction mixture was refluxed for 4.5 h. After addition of water (200 mL), the precipitate generated was filtered and washed with water. The resulting filter cake was dissolved in EtOH under reflux, and then was cooled. The resultant solid was filtered and washed with EtOH to give compound **14** (3.755 g, 59.3%). The filtrate was evaporated to give compound **15** (561.5 mg, 8.1%).

^{1}H NMR (400 MHz, DMSO-d_6) δ 7.41 – 7.39 (m, 2H, 4-H, 7-H), 7.10 – 7.07 (m, 2H, 5-H, 6-H), 4.11 (s, 2H, CH_2).

Benzo[4,5]imidazo[2,1-*b*]thiazol-3(2*H*)-one (15)

To a stirred solution of **14** (2.53 g, 12.15 mmol) in pyridine (10 mL) was added acetic anhydride (2.3 mL, 24.33 mmol), and the reaction mixture was refluxed for 15 min. After cooling, water was added to the reaction mixture and the mixture was stirred at room temperature for 30 min. The resulting solid was filtered and washed with water to give compound **15** (2.103 g, 91.0%).

^{1}H NMR (400 MHz, DMSO-d_6) δ 7.83 (d, 1H, J = 8.0 Hz, 5-H), 7.56 (d, 1H, J = 8.0 Hz, 8-H), 7.34 (t, 1H, J = 8.0 Hz, 7-H), 7.29 (t, 1H, J = 8.0 Hz, 6-H), 4.58 (s, 2H, 2-CH_2); ^{13}C NMR (100 MHz, DMSO-d_6) δ 166.7, 159.9, 150.0, 130.5, 126.2, 124.1, 119.2, 112.5, 40.4.

General procedure for the synthesis of (*Z*)-2-(substituted benzylidene) benzimidazothiazolone derivatives 1 – 13.

A solution of benzimidazothiazolone **15** (100 mg, 0.53 mmol) and an appropriate benzaldehyde (1.0 equiv.) in acetic acid (1 – 2 mL) was heated at 85 °C in the presence of sodium acetate (129 mg, 1.58 mmol) for 15 h – 3 d. After addition of water (20 – 40 mL), the precipitate was filtered, and washed with water, hexane:dichloromethane (5:1), and/or MeOH:H_2O (1:10 – 1:1) to give (*Z*)-2-(substituted benzylidene)benzimidazothiazolone derivatives **1** – **13** as solids in yields of 35.6 – 82.2%.

(*Z*)-2-(4-Hydroxybenzylidene)benzo[4,5]imidazo[2,1-*b*]thiazol-3(2*H*)-one (1)

Solid; reaction time, 15 h; yield, 55.3%; melting point, ≥ 300 ℃; ^{1}H NMR (500 MHz, DMSO-d_6) δ 10.46 (s, 1H, 4′-OH), 7.99 (s, 1H, vinylic H), 7.92 (d, 1H, J = 8.0 Hz, 5-H), 7.64 (d, 1H, J = 8.0 Hz, 8-H), 7.60 (d, 2H, J = 8.5 Hz, 2′-H, 6′-H), 7.38 (t, 1H, J = 8.0 Hz, 7-H), 7.34 (t, 1H, J = 8.0 Hz, 6-H), 6.95 (d, 2H, J = 8.5 Hz, 3′-H, 5′-H); ^{13}C NMR (100 MHz, DMSO-d_6) δ 161.3,

160.1, 154.0, 148.9, 137.2, 133.7, 130.8, 126.2, 124.6, 124.3, 120.8, 119.8, 117.2, 113.0; LRMS (ESI-) *m/z* 293 $(M\text{-}H)^-$.

(*Z*)-2-(3,4-Dihydroxybenzylidene)benzo[4,5]imidazo[2,1-*b*]thiazol-3(2*H*)-one (2)

Solid; reaction time, 15 h; yield, 58.2%; melting point, ≥300 ℃; ^{1}H NMR (500 MHz, DMSO-d_6) δ 10.00 (brs, 1H, OH), 9.57 (brs, 1H, OH), 7.92 (d, 1H, *J* = 8.0 Hz, 5-H), 7.90 (s, 1H, vinylic H), 7.65 (d, 1H, *J* = 8.0 Hz, 8-H), 7.38 (t, 1H, *J* = 8.0 Hz, 7-H), 7.34 (t, 1H, *J* = 8.0 Hz, 6-H), 7.14 (s, 1H, 2′-H), 7.10 (d, 1H, *J* = 8.5 Hz, 6′-H), 6.91 (d, 1H, *J* = 8.5 Hz, 5′-H); ^{13}C NMR (100 MHz, DMSO-d_6) δ 160.1, 154.1, 150.1, 148.9, 146.7, 137.6, 130.8, 126.2, 125.4, 124.6, 124.6, 120.5, 119.8, 117.3, 117.1, 112.9; LRMS (ESI-) *m/z* 309 $(M\text{-}H)^-$.

(*Z*)-2-(2,4-Dihydroxybenzylidene)benzo[4,5]imidazo[2,1-*b*]thiazol-3(2*H*)-one (3)

Solid; reaction time, 15 h; yield, 42.1%; melting point, 263.7 – 268.3 ℃; ^{1}H NMR (500 MHz, DMSO-d_6) δ 12.19 (brs, 1H, 2′-OH), 10.9 (brs, 1H, 4′-OH), 8.42 (s, 1H, vinylic H), 7.59 (d, 1H, *J* = 8.5 Hz, 6′-H), 7.44 – 7.42 (m, 2H, 5-H, 8-H), 7.14 (t, 1H, *J* = 6.5 Hz, 7-H), 7.12 (t, 1H, *J* = 6.5 Hz, 6-H), 6.82 (dd, 1H, *J* = 8.5, 2.5 Hz, 5′-H), 6.77 (d, 1H, *J* = 2.5 Hz, 3′-H); ^{13}C NMR (125 MHz, DMSO-d_6) δ 162.8, 159.3, 156.2, 149.7, 147.6, 130.7, 122.3, 114.1, 112.9, 112.4, 102.5; LRMS (ESI-) *m/z* 309 $(M\text{-}H)^-$.

(*Z*)-2-(4-Hydroxy-3-methoxybenzylidene)benzo[4,5]imidazo[2,1-*b*]thiazol-3(2*H*)-one (4)

Solid; reaction time, 15 h; yield, 61.3%; melting point, 262.3 – 264.0 ℃; ^{1}H NMR (400 MHz, DMSO-d_6) δ 10.13 (s, 1H, OH), 7.96 (s, 1H, vinylic H), 7.88 (d, 1H, *J* = 7.6 Hz, 5-H), 7.61 (d, 1H, *J* = 7.6 Hz, 8-H), 7.34 (t, 1H, *J* = 7.6 Hz, 7-H), 7.30 (t, 1H, *J* = 7.6 Hz, 6-H), 7.25 (d, 1H, *J* = 1.6 Hz, 2′-H), 7.17 (dd, 1H, *J* = 8.4, 1.6 Hz, 6′-H), 6.94 (d, 1H, *J* = 8.4 Hz, 5′-H), 3.82 (s,

3H, OMe); ^{13}C NMR (100 MHz, DMSO-d_6) δ 160.0, 154.0, 150.9, 148.9, 148.7, 137.5, 130.7, 126.2, 125.3, 124.6, 124.6, 120.9, 119.8, 117.0, 115.2, 112.9, 56.3; LRMS (ESI-) *m/z* 323 (M-H)$^-$.

(*Z*)-2-(3-Ethoxy-4-hydroxybenzylidene)benzo[4,5]imidazo[2,1-*b*]thiazol-3(2*H*)-one (5)

Solid; reaction time, 15 h; yield, 45.1%; melting point, 210.7 °C; ^{1}H NMR (400 MHz, DMSO-d_6) δ 10.05 (s, 1H, OH), 7.97 (s, 1H, vinylic H), 7.90 (d, 1H, *J* = 8.0 Hz, 5-H), 7.63 (d, 1H, *J* = 8.0 Hz, 8-H), 7.36 (t, 1H, *J* = 8.0 Hz, 7-H), 7.32 (t, 1H, *J* = 8.0 Hz, 6-H), 7.25 (d, 1H, *J* = 2.0 Hz, 2′-H), 7.18 (dd, 1H, *J* = 8.4, 2.0 Hz, 6′-H), 6.95 (d, 1H, *J* = 8.4 Hz, 5′-H), 4.08 (q, 2H, *J* = 7.2 Hz, CH_2CH_3), 1.34 (t, 3H, *J* = 7.2 Hz, CH_2CH_3); ^{13}C NMR (100 MHz, DMSO-d_6) δ 160.0, 154.0, 151.1, 148.9, 147.9, 137.5, 130.8, 126.2, 125.3, 124.7, 124.6, 120.9, 119.9, 117.1, 116.3, 113.0, 64.6, 15.3; LRMS (ESI-) *m/z* 337 (M-H)$^-$.

(*Z*)-2-(3-Hydroxy-4-methoxybenzylidene)benzo[4,5]imidazo[2,1-*b*]thiazol-3(2*H*)-one (6)

Solid; reaction time, 15 h; yield, 60.8%; melting point, 249.6 – 252.1 °C; ^{1}H NMR (400 MHz, DMSO-d_6) δ 9.60 (s, 1H, OH), 7.90 (s, 1H, vinylic H), 7.89 (d, 1H, *J* = 8.0 Hz, 5-H), 7.62 (d, 1H, *J* = 8.0 Hz, 8-H), 7.36 (t, 1H, *J* = 8.0 Hz, 7-H), 7.31 (t, 1H, *J* = 8.0 Hz, 6-H), 7.18 (d, 1H, *J* = 8.4 Hz, 6′-H), 7.12 (s, 1H, 2′-H), 7.06 (d, 1H, *J* = 8.4 Hz, 5′-H), 3.81 (s, 3H, OMe); ^{13}C NMR (100 MHz, DMSO-d_6) δ 160.0, 154.0, 151.3, 148.9, 147.7, 137.1, 130.8, 126.2, 125.9, 124.9, 124.6, 121.8, 119.8, 116.6, 113.1, 113.0, 56.4; LRMS (ESI-) *m/z* 323 (M-H)$^-$.

(*Z*)-2-(4-Methoxybenzylidene)benzo[4,5]imidazo[2,1-*b*]thiazol-3(2*H*)-one (7)

Solid; reaction time, 15 h; yield, 82.2%; melting point, 239.5 – 241.8 °C; ^{1}H NMR (500 MHz, $CDCl_3$) δ 8.04 (d, 1H, *J* = 8.0 Hz, 5-H), 8.02 (s, 1H, vinylic H), 7.67 (d, 1H, *J* = 8.0 Hz, 8-H),

7.57 (d, 2H, J = 9.0 Hz, 2′-H, 6′-H), 7.38 (t, 1H, J = 8.0 Hz, 7-H), 7.33 (d, 1H, J = 8.0 Hz, 6-H), 7.03 (d, 2H, J = 9.0 Hz, 3′-H, 5′-H), 3.88 (s, 3H, OMe); ^{13}C NMR (100 MHz, $CDCl_3$) δ 162.1, 160.1, 153.8, 149.1, 137.1, 132.8, 130.8, 126.0, 125.7, 124.3, 121.9, 119.7, 115.2, 113.1, 55.8; LRMS (ESI+) *m/z* 309 $(M+H)^+$.

(*Z*)-2-(3,4-Dimethoxybenzylidene)benzo[4,5]imidazo[2,1-*b*]thiazol-3(2*H*)-one (8)

Solid; reaction time, 15 h; yield, 50.2%; melting point, 236.7 – 236.9 °C; ^{1}H NMR (400 MHz, $CDCl_3$) δ 8.01 (d, 1H, J = 7.6 Hz, 5-H), 7.98 (s, 1H, vinylic H), 7.66 (d, 1H, J = 7.6 Hz, 8-H), 7.37 (t, 1H, J = 7.6 Hz, 7-H), 7.32 (t, 1H, J = 7.6 Hz, 6-H), 7.22 (dd, 1H, J = 8.8, 1.6 Hz, 6′-H), 7.07 (d, 1H, J = 1.6 Hz, 2′-H), 6.96 (d, 1H, J = 8.8 Hz, 5′-H), 3.96 (s, 3H, OMe), 3.93 (s, 3H, OMe); ^{13}C NMR (100 MHz, $CDCl_3$) δ 160.0, 153.6, 151.9, 149.7, 148.9, 137.3, 130.7, 126.1, 126.0, 125.2, 124.4, 122.2, 119.7, 113.1, 112.8, 111.7, 56.3, 56.2; LRMS (ESI+) *m/z* 339 $(M+H)^+$.

(*Z*)-2-(2,4-Dimethoxybenzylidene)benzo[4,5]imidazo[2,1-*b*]thiazol-3(2*H*)-one (9)

Solid; reaction time, 15 h; yield, 63.0%; melting point, 250.1 – 257.5 °C; ^{1}H NMR (500 MHz, $CDCl_3$) δ 8.40 (s, 1H, vinylic H), 8.05 (d, 1H, J = 7.5 Hz, 5-H), 7.67 (d, 1H, J = 7.5 Hz, 8-H), 7.52 (d, 1H, J = 8.5 Hz, 6′-H), 7.37 (t, 1H, J = 7.5 Hz, 7-H), 7.32 (t, 1H, J = 7.5 Hz, 6-H), 6.62 (d, 1H, J = 8.5 Hz, 5′-H), 6.48 (s, 1H, 3′-H), 3.92 (s, 3H, OMe), 3.87 (s, 3H, OMe); ^{13}C NMR (100 MHz, $CDCl_3$) δ 164.1, 160.7, 160.4, 154.3, 148.9, 132.9, 131.6, 125.9, 124.1, 121.5, 119.5, 115.4, 113.0, 105.9, 104.5, 98.8, 55.9, 55.9; LRMS (ESI+) *m/z* 339 $(M+H)^+$.

(*Z*)-2-(2-Hydroxybenzylidene)benzo[4,5]imidazo[2,1-*b*]thiazol-3(2*H*)-one (10)

Solid; reaction time, 2 d; yield, 35.6%; melting point, 244.2 – 245.2 °C; ^{1}H NMR (500 MHz,

DMSO-d_6) δ 8.39 (s, 1H, vinylic H), 7.71 (d, 1H, J = 8.0 Hz), 7.63 (t, 1H, J = 8.0 Hz), 7.49 – 7.45 (m, 3H), 7.36 (t, 1H, J = 8.0 Hz), 7.14 – 7.11 (m, 2H); ^{13}C NMR (100 MHz, DMSO-d_6) δ 158.9, 153.7, 146.4, 146.0, 144.5, 136.0, 133.2, 129.2, 125.6, 123.2, 122.3, 120.6, 119.8, 118.9, 116.9, 111.6.

(*Z*)-2-(3,4,5-Trimethoxybenzylidene)benzo[4,5]imidazo[2,1-*b*]thiazol-3(2*H*)-one (11)

Solid; reaction time, 3 d; yield, 78.4%; melting point, 215.1 °C; ^{1}H NMR (500 MHz, $CDCl_3$) *δ* 8.03 (d, 1H, J = 7.5 Hz, 5-H), 7.98 (s, 1H, vinylic H), 7.68 (d, 1H, J = 7.5 Hz, 8-H), 7.39 (t, 1H, J = 7.5 Hz, 7-H), 7.34 (t, 1H, J = 7.5 Hz, 6-H), 6.84 (s, 2H, 2′-H ,6′-H), 3.94 (s, 6H, 2×OMe), 3.93 (s, 3H, OMe); ^{13}C NMR (100 MHz, $CDCl_3$) *δ* 159.7, 153.9, 153.4, 149.0, 140.9, 137.2, 130.6, 128.5, 126.2, 124.5, 124.1, 119.9, 113.1, 107.9, 61.3, 56.5; LRMS (ESI+) *m/z* 369 $(M+H)^+$.

(*Z*)-2-(4-Hydroxy-3,5-dimethoxybenzylidene)benzo[4,5]imidazo[2,1-*b*]thiazol-3(2*H*)-one (12)

Solid; reaction time, 15 h; yield, 68.4%; melting point, 261.9 – 269.1 °C; ^{1}H NMR (400 MHz, DMSO-d_6) *δ* 9.50 (s, 1H, OH), 7.95 (s, 1H, vinylic H), 7.89 (d, 1H, J = 7.6 Hz, 5-H), 7.62 (d, 1H, J = 7.6 Hz, 8-H), 7.34 (t, 1H, J = 7.6 Hz, 7-H), 7.30 (t, 1H, J = 7.6 Hz, 6-H), 6.97 (s, 2H, 2′-H, 6′-H), 3.82 (s, 6H, 2×OMe); ^{13}C NMR (100 MHz, DMSO-d_6) *δ* 159.9, 153.9, 149.0, 149.0, 140.1, 137.7, 130.8, 126.2, 124.6, 123.5, 121.3, 119.9, 112.9, 109.0, 56.8; LRMS (ESI-) *m/z* 353 $(M-H)^-$.

(*Z*)-2-(3-Bromo-4-hydroxybenzylidene)benzo[4,5]imidazo[2,1-*b*]thiazol-3(2*H*)-one (13)

Solid; reaction time, 2 d; yield, 68.7%; melting point, 293.7 – 297.0 °C; ^{1}H NMR (400 MHz,

DMSO-d_6) δ 11.31 (s, 1H, OH), 7.96 (s, 1H, vinylic H), 7.92 – 7.89 (m, 2H, 5-H, 2′-H), 7.63 (d, 1H, J = 8.0 Hz, 8-H), 7.56 (d, 1H, J = 8.4 Hz, 6′-H), 7.36 (t, 1H, J = 8.0 Hz, 7-H), 7.32 (t, 1H, J = 8.0 Hz, 6-H), 7.10 (d, 1H, J = 8.4 Hz, 5′-H); ^{13}C NMR (100 MHz, DMSO-d_6) δ 159.8, 157.6, 153.8, 148.9, 136.8, 135.6, 131.4, 130.8, 126.3, 125.9, 124.7, 122.6, 119.9, 117.7, 113.0, 111.0; LRMS (ESI-) *m/z* 371 $(M-H)^-$.
